# Ear, Nose, and Throat (ENT) Emergency Training in Undergraduate Medical Education: Global Status, Gaps, and Curriculum Reform Priorities

**DOI:** 10.7759/cureus.97043

**Published:** 2025-11-17

**Authors:** Al Mahdin Ornob Miah, Filippo Cainelli, Vikas Acharya

**Affiliations:** 1 Hospital Medicine, Barts Health Trust, London, GBR; 2 Medicine and Surgery, St George’s Hospital, London, GBR; 3 ENT Surgery, Imperial College Healthcare NHS Trust, London, GBR

**Keywords:** competency based education, curriculum reform, ent emergencies, otolaryngology education, simulation-based learning, undergraduate medical training

## Abstract

Ear, Nose, and Throat (ENT) emergencies, such as airway obstruction, severe epistaxis, and foreign body aspiration, are frequent and potentially life-threatening conditions commonly encountered in clinical practice. Despite their prevalence, emergency training in ENT remains insufficient in undergraduate medical education worldwide. This study explores the current status of ENT emergency education, highlighting key gaps and proposing strategies for curriculum reform. Evidence indicates that medical students often graduate with limited competence in managing acute ENT presentations due to short clinical rotations, lack of simulation-based practice, and minimal assessment of procedural skills. These deficiencies are particularly pronounced in low- and middle-income countries, where limited resources and inadequate faculty development further hinder learning outcomes. Given that ENT emergencies account for a significant proportion of emergency department visits globally, improving undergraduate preparedness is essential to ensure safe and effective patient care. Integrating simulation-based learning, standardised national curricula, and e-learning platforms can enhance both theoretical understanding and practical competence. Developing competency-based frameworks and faculty training programs will further ensure sustainability and equity in education. This paper emphasises the need for coordinated global efforts to reform ENT emergency teaching, promoting a generation of doctors capable of responding confidently and effectively to critical otolaryngologic emergencies.

## Introduction and background

ENT emergency competence refers to the integrated ability to apply essential knowledge, procedural skills, and rapid clinical decision-making to recognise, prioritise, and manage life-threatening or urgent ear, nose, and throat conditions effectively [1]. ENT problems represent the third largest surgical specialty and are commonly encountered in both emergency departments and primary care. Epidemiological studies illustrate the burden: in a five-year retrospective study of 38,793 patients at a tertiary hospital in India, epistaxis alone accounted for 25.58% of all ENT emergencies. The same study showed earache and ear discharge were among the next most frequent presentations [1]. In southern Europe, epistaxis made up about 3.31% of all emergency department (ED) visits (≈1 in 30), in a study spanning 2009-2015 [2]. Another cross-sectional hospital study in Belgium evaluated 1,296 ENT emergency visits where epistaxis was the most frequent diagnosis, followed by infections of the pharynx and tonsils; nasal fractures and vertigo were also common [1]. Despite this burden, ENT receives only modest representation in undergraduate medical curricula. According to surveys and reports, many medical students and junior doctors report being under-prepared to handle common ENT emergencies [3]. In the UK, over 42% of newly qualified doctors stated that they felt unready for their first clinical post. Medical schools often provide very limited clinical exposure to ENT, rotations are short or optional, and formal assessments of ENT emergency competence are frequently absent [4].

However, lack of exposure is not the sole cause of underpreparedness. Other contributing factors include curricular overcrowding (competition for time among many specialties), institutional prioritisation of those viewed as higher mortality or higher profile (e.g., cardiology, surgery), student perceptions that ENT is a niche rather than broadly relevant, and variation in faculty and resource availability. Assessment frameworks are inconsistent, and many medical schools lack clearly defined competencies for ENT emergencies. Given the frequency and diversity of ENT emergencies-ranging from epistaxis, peritonsillar abscess, airway compromise, foreign body airway obstruction, and acute ear infections [5], graduates must possess not only theoretical knowledge but also procedural skills and decision-making confidence. This review, therefore, examines the global and UK status of ENT emergency training in undergraduate medical education, identifies gaps in current provision (clinical exposure, assessment, standardisation), and proposes evidence-based recommendations to enhance curriculum design so that future physicians are competent and confident in managing ENT emergencies.

## Review

Methodology

**Figure 1:**
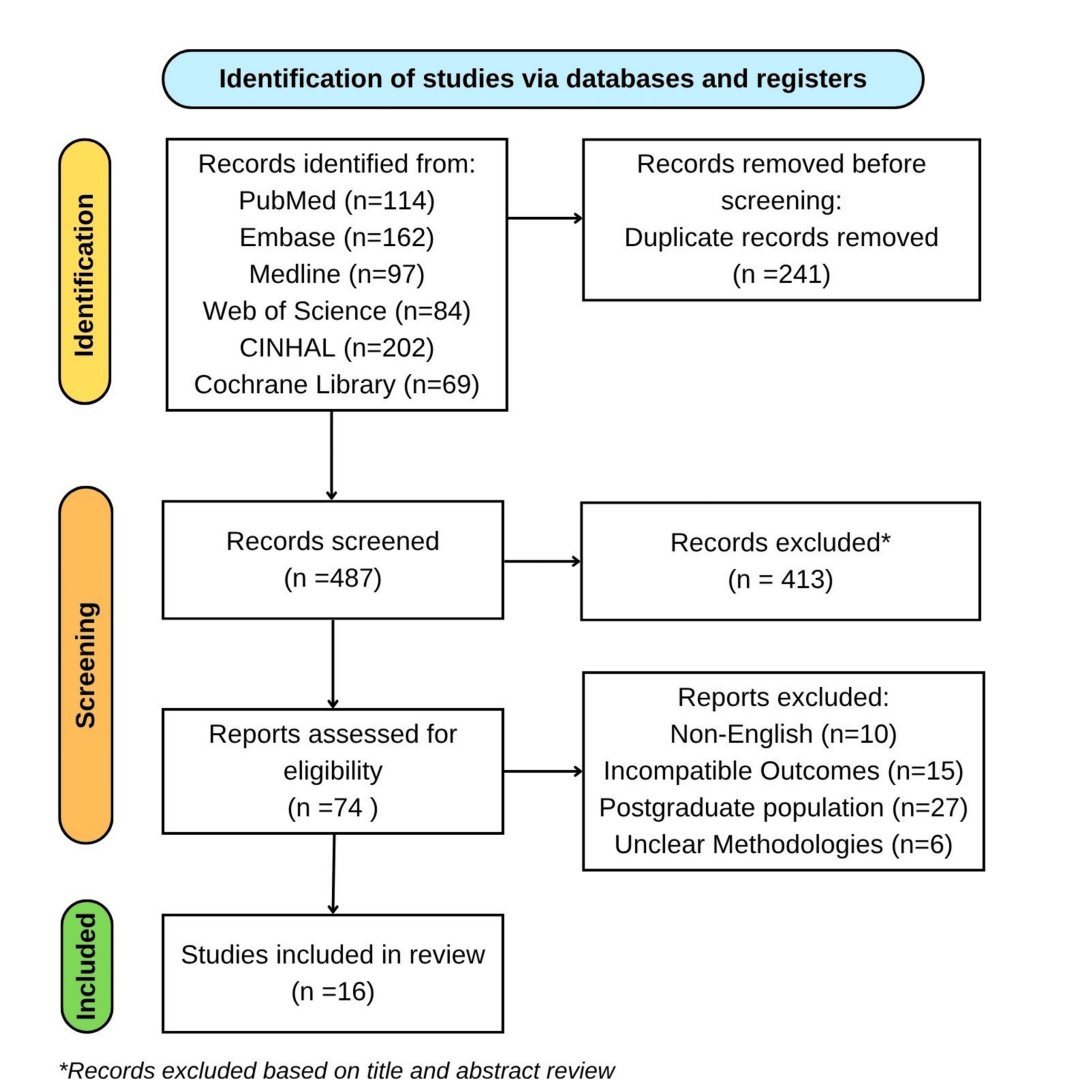
PRISMA Flow Diagram

A narrative literature review was undertaken to evaluate the global and UK status of ENT emergency training within undergraduate medical education. Following Preferred Reporting Items for Systematic Reviews and Meta-Analyses (PRISMA) principles (Figure 1), a comprehensive search was conducted across PubMed, Medical Literature Analysis and Retrieval System Online (MEDLINE), Excerpta Medica Database (EMBASE), Cumulative Index to Nursing and Allied Health Literature (CINAHL), Web of Science, and the Cochrane Library for studies published between January 2000 and April 2025. The Boolean search strategy used was: (“undergraduate medical education” OR “medical students”) AND (“otolaryngology” OR “ENT”) AND (“emergency” OR “acute care” OR “airway” OR “epistaxis” OR “foreign body”) AND (“training” OR “curriculum” OR “simulation” OR “competence”). Studies were included if they explored ENT teaching, clinical exposure, curriculum design, or simulation-based learning at the undergraduate level, while those focused solely on postgraduate education, editorials, or lacking primary data were excluded. From an initial 728 articles, 241 duplicates were removed. The remaining 487 records were screened, of which 16 proceeded through title, abstract, and full-text review to be included in the final analysis. Two reviewers independently screened and extracted data, achieving strong inter-rater reliability (Cohen’s κ = 0.86). Extracted information was summarised by country, study design, outcomes, and evidence level. Themes were derived through inductive coding to identify patterns relating to curriculum structure, student competence, simulation practices, and barriers to training, forming the analytical basis for this review’s findings and recommendations.

Results

Current Global Literature

Otolaryngologic conditions are frequently encountered in primary care and emergency settings, yet they remain underrepresented in undergraduate medical education (UME). Globally, literature consistently highlights the inadequacy of ENT education within medical school curricula, particularly regarding emergency management competencies. Fung et al. explored the representation of otolaryngology in undergraduate medical education (UME) across Canadian medical schools, revealing significant variability and overall limited exposure [6]. Their survey of undergraduate otolaryngology directors, family and emergency medicine postgraduate directors, and community otolaryngologists indicated that while otolaryngologic issues are common in primary care, structured teaching time dedicated to this field remains minimal and inconsistent. Their findings revealed that there is a large variation in the quantity of otolaryngology teaching in UME. Knowledge of otolaryngology is formally evaluated at half of the responding programs, although skills in otolaryngology are rarely tested. Clerkship rotations are not uniformly offered, and the length of these placements is limited. Opportunities in postgraduate training for formal education in otolaryngology are rare. Several important topics that are not uniformly taught include sudden sensorineural hearing loss, sleep apnea, and nasal trauma. The medical community needs standardisation for two important clinical skills that encompass treatment methods for benign paroxysmal positional vertigo and epistaxis conditions. The absence of standardised teaching shows that ENT emergency competency receives insufficient attention from the healthcare system.

The study conducted by Rosvall et al. examined Canadian medical student' readiness to treat ENT conditions by revealing major shortcomings in their clinical practical experience and readiness to manage ENT disorders [7]. The survey results showed that 46 out of 87 fourth-year students from two Canadian universities received no clinical otolaryngology-head and neck surgery (OHNS) experience, and 29 students rated their OHNS education as insufficient. Data showed concerning results regarding the inability of medical students to recognise immediate referral conditions in ear-nose-throat medicine, which remains crucial for emergency management competencies. Research results showed a direct link between OHNS exposure time and student competence in treating ENT disorders (r = 0.267, P = .012). This research highlighted the importance of intensive OHNS training in medical undergraduate curricula because it enables future doctors to treat ENT emergencies properly [7].

Pasick et al. performed a multi-institutional assessment of basic otolaryngology knowledge for medical students across nine U.S. allopathic institutions. The validated multiple-choice quiz contained nine questions to evaluate students’ theoretical knowledge, along with their comfort in treating otolaryngologic conditions, plus their exposure to otolaryngology and faculty-directed training on head and neck examination procedures. Analysis of 547 student responses revealed a strong positive correlation between otolaryngology exposure and knowledge (P < .001, R² = 0.284), as well as between knowledge and comfort in managing ENT conditions (P < .001, R² = 0.266). Students intending to enter otolaryngology had higher scores (P = .002), higher comfort levels in managing otolaryngologic cases (P < .001), and higher comfort levels performing the head and neck examination, compared with students intending to enter primary care or another surgical specialty [8]. Clinical students in third and fourth years entering any surgical specialty (including otolaryngology), more often than students entering primary care (P = .007), recognised the obturator as the instrument used to guide the insertion of the tracheotomy tube. Additionally, the number of times the head and neck examination was taught correlated positively with comfort in performing the examination.

Importantly, the landmark systematic review by Ishman et al. conducted a qualitative synthesis of 47 studies and provided one of the most comprehensive overviews of ENT education worldwide. Their findings revealed that although otolaryngology-related complaints account for approximately 25% of all primary care consultations, ENT rotations were not mandatory in most medical schools across the United States, the United Kingdom, and Canada [9]. Moreover, Ishman et al. found that most studies describing ENT education were low-level evidence (levels 3 or 4), predominantly needs assessments, curriculum descriptions, or educational method evaluations, underscoring the scarcity of high-quality, outcome-driven research in this domain [9].

The growing role of innovative teaching methods in undergraduate ENT education is well highlighted by recent studies, particularly the works of MK et al. and Fung [10-11]. MK et al.conducted a prospective interventional study involving 60 interns to compare traditional lecture-based teaching with simulation-based hybrid training for critical ENT emergencies, including tracheostomy management, nasogastric tube insertion, and epistaxis control [10] Their findings demonstrated significant improvements in both knowledge and psychomotor skills among those who received hands-on simulation training, with objective assessments confirming superior performance in emergency scenarios (P < 0.05). The study confirmed that simulation effectively improves both theoretical knowledge and procedural capability, thus demonstrating its essential worth for teaching students how to respond to urgent situations in ENT medicine [10]. Fung presented a synthesis of OHNS curriculum representation analysis from the past decade with proposed improvement strategies [11]. According to him, e-learning works well for teaching essential clinical knowledge and procedural skills; however, it does not perform well with complex spatial anatomy simulation, allowing evolving immersive practical learning opportunities. Standardised OHNS learning objectives should be established with attention to specific student learning methods and technological limitations.

**Figure 2:**
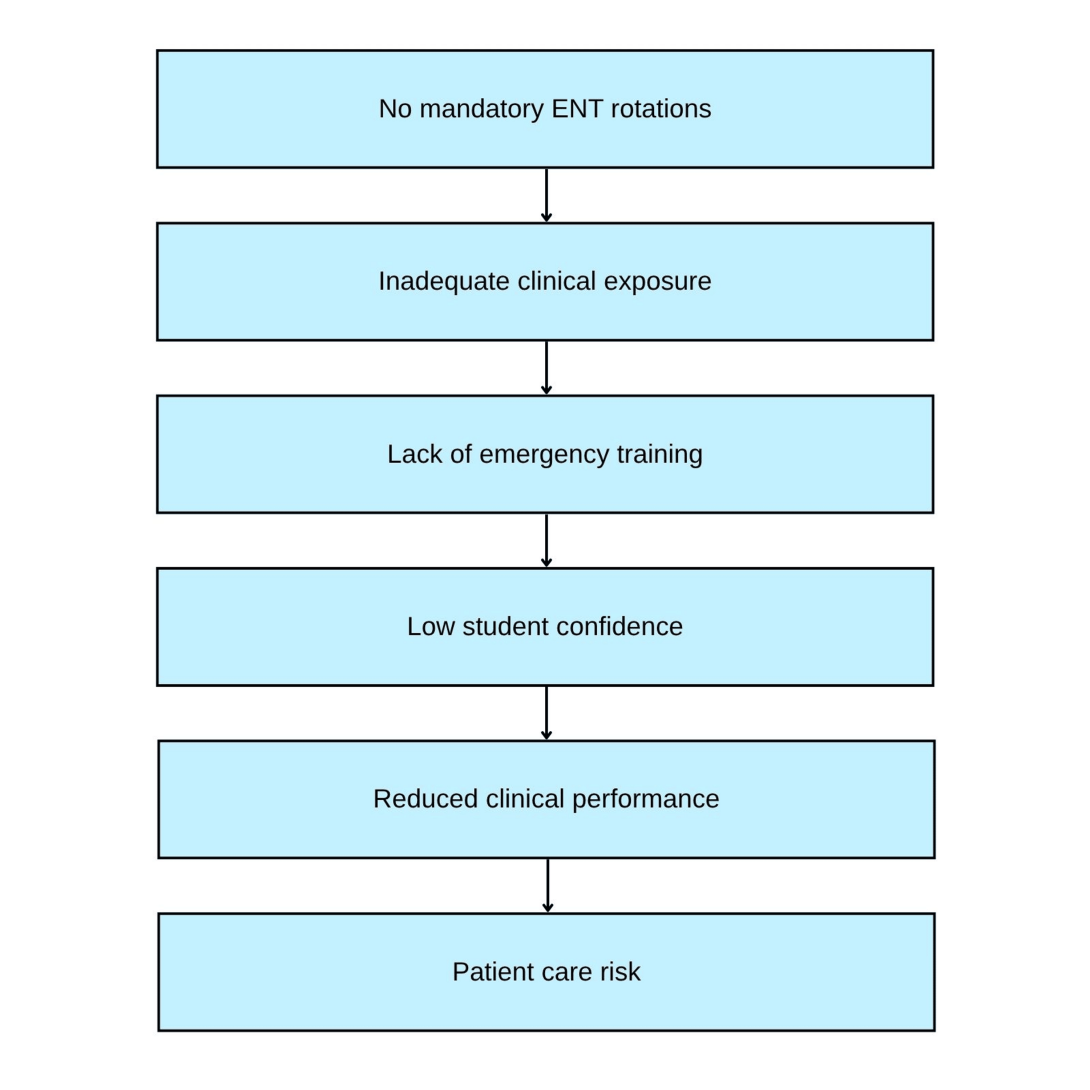
Flowchart: ENT Education Gaps Leading to Competency Deficits

These critical gaps in ENT education research are summarised in Figure 2.

Current UK Literature

The current provision of undergraduate ENT education in the United Kingdom has been consistently shown to be inadequate, fragmented, and insufficiently aligned with the clinical demands faced by new medical graduates. Despite longstanding concern, multiple studies highlight that minimal progress has been made over the past two decades in strengthening ENT education across UK medical schools. Khan et al. undertook a survey evaluating ENT training across UK institutions and found that while slightly more than half of the medical schools provided a compulsory ENT placement, ten schools did not offer any mandatory ENT clinical experience. Where placements were available, the average duration was a mere eight days [12]. Alarmingly, only 38% of students reported satisfaction with their ENT training, and ENT consultants widely perceived that newly qualified doctors lacked basic competence in managing common ENT conditions. These findings echoed earlier concerns raised by Mace et al., who found that 22% of medical schools had no compulsory ENT attachment and that many ENT rotations were merged with dermatology, ophthalmology, or neurology placements, reducing focused ENT exposure to as little as one and a half weeks on average [13]. Furthermore, formal assessments of ENT knowledge or clinical skills were absent in 42% of cases, reflecting a concerning lack of accountability in verifying student competence [13].

Recent qualitative work by Patel et al. has added depth to these findings by exploring perceptions from a range of stakeholders, including students, GPs, ENT surgeons, and curriculum developers [14]. Their study identified multiple barriers to effective ENT education, including student-related factors (e.g., lack of perceived relevance), teacher-related challenges (e.g., shortage of trained faculty), and environmental limitations (e.g., time constraints within the curriculum). They proposed diversifying ENT teaching through greater use of simulation, e-learning, and general practice placements to enhance skill acquisition and reinforce the real-world applicability of ENT competencies.

Graduates’ perspectives on their preparedness for ENT practice have further confirmed the critical need for reform. Powell et al. (2011) surveyed newly qualified doctors and found that the mean ENT exposure during undergraduate training amounted to approximately five days of clinical experience [15]. Notably, 15.8% of respondents reported no formal ENT department experience at all. Over 65% expressed a desire for more ENT training, and clinical confidence in ENT was significantly lower compared to competencies in specialties like cardiology (P < 0.001). These results highlight not only a shortfall in training but also its downstream effects on junior doctor performance and patient care.

Sharma et al. extended these concerns into the accident and emergency (A&E) setting, surveying senior house officers working in emergency departments. They found that while 90% of respondents recognised the value of undergraduate ENT education for A&E practice, 75% felt their training had been inadequate, and 45% reported no postgraduate ENT teaching during their early clinical careers [16]. This underscores the critical role of undergraduate ENT exposure in preparing doctors for frontline specialties, where ENT emergencies such as airway compromise or severe epistaxis are common.

Concerns about ENT training have also been echoed within general practice. Clamp et al. surveyed 500 GPs in southwest England and found that although most had received about two weeks of undergraduate ENT training, the majority deemed this insufficient [17]. Furthermore, postgraduate ENT training remained highly variable, with almost half of the respondents considering their ongoing ENT education inadequate. Importantly, 75% of GPs expressed a desire for additional ENT training, underlining the persistent relevance of ENT skills in primary care practice. Additional insights into the experiential aspects of ENT education have been provided by [18-19]. Lee et al. evaluated student perceptions of operating theatre attendance during ENT rotations and found that while 60% of students felt their educational expectations were met, satisfaction with theatre teaching was modest, reflecting a need for more structured learning objectives during surgical exposure [18]. Hajioff et al., focusing on outpatient clinic teaching, demonstrated that the presence of students did not negatively impact patient satisfaction or clinic efficiency. Importantly, students reported higher satisfaction when given opportunities to interact directly with patients, emphasising the value of active clinical involvement in ENT education [19].

Recent studies increasingly disaggregate emergency ENT education from general ENT curriculum findings. Simulation-based training, in particular, shows promise in improving competence for emergency scenarios: for example, a prospective interventional study in India found that interns who received hands-on simulation training on tracheostomy, epistaxis, and nasogastric insertion significantly outperformed peers who received only lecture-based instruction (P < 0.05) in simulated emergency tasks [10] In addition, a 2023 simulation-based workshop for otolaryngologic emergencies demonstrated marked reductions in participant anxiety and increased confidence in airway and foreign-body management immediately post-training [20]. A 2024 study from Rwanda implemented a low-cost simulation curriculum (cricothyrotomy, foreign-body removal, epistaxis) in a resource-limited context, showing improved knowledge and self-reported confidence among participating students [21]. These findings underscore that targeted simulation can bolster emergency preparedness, even in constrained settings. However, most other studies still report general deficits across ENT curricula and do not separate emergency training explicitly. Therefore, the specificity and consistency of emergency‐focused content remain lacking, making comparisons across institutions difficult. However, most other studies still report general deficits across ENT curricula and do not separate emergency training explicitly. Therefore, the specificity and consistency of emergency‐focused content remain lacking, making comparisons across institutions difficult. These comparative findings are summarised in Table 1, which provides an overview of ENT rotation requirements, average duration, and student satisfaction across major regions.

**Table 1:** Comparison of ENT Rotation Lengths and Requirements

Country	Mandatory ENT Rotation	Average Duration	ENT in Exams	Student Satisfaction
UK	52%	5–8 days	58%	38%
Canada	<50%	Varies	50%	Low (varies)
USA	<50%	Varies	50%	50%

Gaps in Current Undergraduate ENT Education

Despite increased recognition of the importance of ENT in general and emergency medical practice, significant gaps persist in undergraduate medical education, both globally and particularly in the UK. Numerous studies have highlighted that ENT remains grossly underrepresented within the medical curriculum, often receiving only minimal clinical time [12]. Many medical schools still do not offer mandatory ENT attachments, and where rotations do exist, they are typically short, poorly structured, and often combined with other specialties, diluting specialty-specific learning [13, 17]. Even where clinical exposure is available, a lack of formal competency-based assessment means that students may graduate without verifiable proficiency in managing common ENT emergencies [14-15, 22]. The ability of students to handle conditions affecting the ears, nose, and throat area stands lower than other fundamental medical practices, according to consistent research [16-17]. New medical professionals and general practitioners frequently express negative opinions about undergraduate and postgraduate ENT training programs. The combination of traditional educational practices lacks effective student engagement and proper psychomotor skills development needed for treating ENT emergencies because simulation training and practical patient care opportunities remain underused [10-11]. Students appreciate outpatient clinics and theatre attendance, but these experiences often miss structured educational plans that optimise learning [19]. The lack of standardised ENT curricula across all UK medical schools produces varying quality and educational content and assessment methods in teaching, which hampers uniform graduate competencies [9, 23].

Structural and Institutional Barriers to Reform

Despite recognition of these gaps, substantial barriers have impeded curricular reform. A survey of young otolaryngologists globally showed that cost, limited access, and time constraints are the most persistent obstacles to implementing simulation-based training in ENT (65.5%, 49.2%, and 25.5% respectively) [24]. The same survey prioritised emergency procedures (tracheotomy, cricothyroidotomy, rigid bronchoscopy) as critical simulation targets, yet noted limited availability of simulation tools for these [24]. Another obstacle lies in institutional inertia and competition between curricula: smaller specialties such as ENT tend to lose in the competition over the limited hours of teaching. Additionally, training ENT faculty or simulation facilities is also not present in most medical schools and in low and middle-income countries. Equity problems contribute to the discrimination, as simulation and structured training can be applied more easily at schools that have better resources. The introduction of low-cost simulation models in the low-resource environment is an evidence-based solution (e.g., Rwanda study, above) but needs leadership and funding. Lastly, emergency ENT competence is not mandated by national or accrediting bodies and, therefore, change is not mandatory but optional in most institutions [21]. This may be indicated by the fact that emergency-specific training in ENT is poorly developed, despite the potential that has been shown by simulation and curriculum advancement. Structural barriers have been used to hold back progress such as resource limitation, conflicting curricular priorities, and institutional mandates. These systemic problems have to be mitigated by reforms to increase the pace of change, namely, national guidelines, common infrastructure in simulation, open access models, and core emergency ENT competency requirements. The attainment of a material change in undergraduate ENT emergency competence can only be accomplished with valuable and lasting gain, with the distinction between emergency-centered and general ENT teaching, and the removal of impediments to progress through the attainment of undergraduate ENT emergency competence.

Recommendations for Curriculum Improvement

There should be a complete overhaul of the teaching of ENT in undergraduate medical universities to curb the existing gaps. All medical schools should determine independent specialised ENT rotations exceeding two weeks for students to become well acquainted with all the necessary emergency cases related to ears, nose, and throat [12, 14]. The rotations must avoid merging with unrelated specialties since this dilutes learning specific to ENT skills. A standardised ENT curriculum must be created at a national level, which will outline standard competence guidelines that medical graduates need to fulfill for professional certification [11]. Formal assessments that test ENT knowledge and clinical abilities should incorporate objective structured clinical examinations (OSCEs) and structured clinical examinations to objectively prove [13, 15]. Simulation-based learning, which focuses on vital emergency procedures such as tracheotomy care, along with epistaxis management and airway management, plays a vital role in training mental and physical skills without endangerment [10, 11]. The structure for integrating ENT teaching into outpatient facilities should prioritise student engagement while permitting hands-on practice to achieve learning and patient contentment goals [19]. E-learning modules should be developed together with team-based learning approaches because they will reinforce theoretical concepts while adding flexibility to traditional bedside teaching [9, 11]. The expansion of ENT training must reach general practice environments since these facilities provide exposure to frequent presentations related to the ears and related systems [14, 17]. Such thorough changes to medical education will resolve existing gaps to produce doctors who handle ENT treatments securely with skill and confidence.

In addition to high-risk scenarios, simulation-based training should be extended to include common ENT presentations frequently encountered in emergency settings. These include conditions such as acute otitis media, foreign body removal (ear, nose, or throat), anterior and posterior epistaxis, peritonsillar abscess drainage, and the initial assessment of airway compromise. A targeted simulation approach covering these common presentations can enhance clinical preparedness and bridge gaps in procedural competence among undergraduates [10]. By recreating realistic, time-pressured scenarios, students can develop decision-making skills and confidence in managing ENT emergencies in a safe environment. Evidence from prospective interventional studies supports the superior performance outcomes associated with simulation compared to traditional lecture-based teaching [10]. E-learning modules may also be used to reinforce theoretical knowledge before or after simulation sessions, providing a blended approach to maximise learning outcomes [11, 22].

Future studies need to concentrate on longitudinal outcome studies involving retention and transfer of ENT emergency skills to clinical practice and multi-institutional trials involving standardised curricula, and cost-effective studies comparing traditional and blended training models. Moreover, the next important step is to create faculty development models in ENT pedagogy and evaluate their effectiveness in the context of improving the quality of teaching. Out of these pilot projects, e.g., national simulation courses by ENT UK and inter-professional airway management workshops, can be considered successful evidence of scalable designs of these innovations in other countries around the world.

## Conclusions

This review underlines the longstanding gaps in undergraduate education in ENT worldwide and the UK. Even though the prevalence of ENT-related conditions is so high in clinical practice, the training of ENT is still underrepresented, and there is a lack of clinical exposure, as well as standardised training, and poor assessment of competency. The educational failures are evident in the fact that graduates state that they feel low confidence when handling ENT emergencies. Such promising solutions as e-learning, structured outpatient teaching, and simulation-based training have been found to be effective. Such gaps require a total change, which would include obligatory ENT rotations, competence-based curricula, formal testing, and increased clinical and simulated experiences. The need to strengthen the education on ENT is only necessary so that in the future, physicians will be more prepared to handle the common and urgent ENT conditions with a high degree of competence and confidence in different healthcare environments.

## References

[1] Raj A, Wadhwa V, Jain A (2019). Epidemiological profile of ent emergencies: our experience. Indian J Otolaryngol Head Neck Surg.

[2] Reis LR, Correia F, Castelhano L, Escada P (2018). Epidemiology of epistaxis in the emergency department of a southern European tertiary care hospital. Acta Otorrinolaringol Esp (Engl Ed).

[3] Cave J, Goldacre M, Lambert T, Woolf K, Jones A, Dacre J (2007). Newly qualified doctors’ views about whether their medical school had trained them well: questionnaire surveys. BMC Med Educ.

[4] Biswas D, Rafferty A, Jassar P (2009). Night emergency cover for ENT in England: a national survey. J Laryngol Otol.

[5] Sreekanth G, Novshaba Novshaba, Reddy LS, Bhushan IP (2022). An overview of emergencies in otorhinolaryngology at a tertiary care centre, Telangana. Indian J Otolaryngol Head Neck Surg.

[6] Beyea JA, Wong E, Bromwich M, Weston WW, Fung K (2008). Evaluation of a particle repositioning maneuver web-based teaching module. Laryngoscope.

[7] Rosvall BR, Singer Z, Fung K, Chin CJ (2020). Do medical students receive adequate otolaryngology training? A Canadian perspective. Ann Otol Rhinol Laryngol.

[8] Pasick LJ, Benito D, Zapanta P, Sataloff RT (2019). Assessing medical student basic otolaryngology knowledge: a multi-institutional study. Ear Nose Throat J.

[9] Ishman SL, Stewart CM, Senser E (2015). Qualitative synthesis and systematic review of otolaryngology in undergraduate medical education. Laryngoscope.

[10] Mk G, Saldanha M, Bhat VS, A R, Vincent MJ, Ravikumar A (2023). Simulation-based training in ear, nose and throat skills and emergencies. Braz J Otorhinolaryngol.

[11] Fung K (2015). Otolaryngology--head and neck surgery in undergraduate medical education: advances and innovations. Laryngoscope.

[12] Khan MM, Saeed SR (2012). Provision of undergraduate otorhinolaryngology teaching within General Medical Council approved UK medical schools: what is current practice?. J Laryngol Otol.

[13] Mace AD, Narula AA (2004). Survey of current undergraduate otolaryngology training in the United Kingdom. J Laryngol Otol.

[14] Patel B, Saeed SR, Smith S (2021). The provision of ENT teaching in the undergraduate medical curriculum: a review and recommendations. J Laryngol Otol.

[15] Powell J, Cooles FA, Carrie S, Paleri V (2011). Is undergraduate medical education working for ENT surgery? A survey of UK medical school graduates. J Laryngol Otol.

[16] Sharma A, Machen K, Clarke B, Howard D (2006). Is undergraduate otorhinolaryngology teaching relevant to junior doctors working in accident and emergency departments?. J Laryngol Otol.

[17] Clamp PJ, Gunasekaran S, Pothier DD, Saunders MW (2007). ENT in general practice: training, experience and referral rates. J Laryngol Otol.

[18] Lee MS, Montague ML, Hussain SS (2005). Student-perceived benefit from otolaryngology theatre attendance. J Laryngol Otol.

[19] Hajioff D, Birchall M (1999). Medical students in ENT outpatient clinics: appointment times, patient satisfaction and student satisfaction. Med Educ.

[20] La Monte OA, Lee JH, Soliman SI (2023). Simulation-based workshop for emergency preparedness in otolaryngology. Laryngoscope Investig Otolaryngol.

[21] Nuss S, Wittenberg R, Salano V (2024). Otolaryngology simulation curriculum development and evaluation for medical education in Rwanda. OTO Open.

[22] Grose E, Best C, Liao G (2021). LearnENT: the development of a free open access medical education app in otolaryngology-head and neck surgery. J Surg Educ.

[23] Ferguson GR, Bacila IA, Swamy M (2016). Does current provision of undergraduate education prepare UK medical students in ENT? A systematic literature review. BMJ Open.

[24] Favier V, Ayad T, Blanc F, Fakhry N, Andersen SA (2021). Use of simulation-based training of surgical technical skills among ENTs: an international YO-IFOS survey. Eur Arch Otorhinolaryngol.

